# ZnO-nanoparticles and stage-based drought tolerance in wheat (*Triticum aestivum* L.): effect on morpho-physiology, nutrients uptake, grain yield and quality

**DOI:** 10.1038/s41598-025-89718-2

**Published:** 2025-02-13

**Authors:** Muhammad Aown Sammar Raza, Faqeer Muhammad, Muhammad Farooq, Muhammad Usman Aslam, Naseem Akhter, Monika Toleikienė, Manal Abdulaziz Binobead, M Ajmal Ali, Muhammad Rizwan, Rashid Iqbal

**Affiliations:** 1 Department of Agronomy, Faculty of Agriculture and Environment, The Islamia University of Bahawalpur, Bahawalpur, 63100 Pakistan; 2 Department of Environmental Sciences and Engineering, Government College University Faisalabad, Allama Iqbal Road, Faisalabad, 38000 Pakistan; 3 Department of Chemistry, Government Sadiq College Women University, Bahawalpur, 63100 Pakistan; 4 Institute of Agriculture, Lithuanian Research Centre for Agriculture and Forestry, Instituo Al. 1, LT- 58344, Akademija, Kedainiai, Lithuania; 5 Department of Food Science and Nutrition, College of Agriculture Food Science, King Saud University, Riyadh, 11451 Saudi Arabia; 6 Department of Botany and Microbiology, College of Science, King Saud University, Riyadh, 11451 Saudi Arabia; 7 Institute of Crop Science and Resource Conservation (INRES), University of Bonn, 53115 Bonn, Germany; 8 Department of Life Sciences, Western Caspian University, Baku, Azerbaijan

**Keywords:** Zinc-oxide nanoparticles, Tillering stage, Grain filling stage, Water stress, Leaf gas exchange, Plant sciences, Environmental sciences

## Abstract

Drought-stressed and zinc-deficient soils are major contributors to reduced wheat yields and low-quality grains, especially in semi-arid regions of the world. Zinc-oxide nanoparticles (ZnO-NPs) are adept enough to avoid these losses if applied under the right dose at the right growth stage of many crops including wheat (*Triticum aestivum* L.). Therefore, a pot experiment was conducted with four levels of ZnO-NPs (0, 50, 100 and 150 ppm), and drought imposed at tillering (D_1_) and grain filling (D_2_) stages, considering normal irrigation as control (D_0_), to explore interactive effects of ZnO-NPs and drought episodes on growth, eco-physiology, yield, and grain quality of wheat. The results depicted dose and growth stage-dependent variations in all recorded parameters. ZnO-NPs (150 ppm) significantly increased the number of grains (12.5%), grain weight (12.4%), total yield (25.5%), and zinc contents (58.6%) when the crop was exposed to drought stress at tillering stage, compared to the control treatment. Likewise, drought at grain filling stage with ZnO-NPs (150 ppm) significantly enhanced plant height, spike length, biomass, zinc contents, and grain protein by 15.5%, 3.2%, 16.7%, 100.0%, and 53.8%, respectively, when compared with control treatment. Thus, ZnO-NPs emerged as a potential drought alleviator and yield-oriented safe nano-fertilizer for wheat in semi-arid regions facing irrigation challenges.

## Introduction

Drought among all other abiotic stresses is meticulous as one of the top listed environmental issue restricting different plant growth aspects and causing more severe yield losses leading to serious food scarcity^1^. The population figure across the globe is estimated to rise by about 9–10 billion or even more masses up to the middle of the present century. Contrariwise, exact in the same pace of time, the droughts may lead to serious food insecurity that will probably get worse under anticipated global climate changes^2–4^. Thus, this upsetting global masses’ upsurge, demands increased food production to meet global food requirements. Global extreme weather disasters and increased droughts put global food security at high risk which is getting agitated further by ever-proliferating global residents and their feeding challenges^5^. Considering all the facts, water for crop irrigation could be branded as the highest curbing natural resource in agroecosystems of arid and semi-arid backgrounds^6^. Among the prime sources, drought is one of the main sources of insufficient agronomic productions in semi-arid and arid regions across the globe^7^. For maximum parts of these regions, drought is a spartan restraint of plant growth, development, and productivity^8^. Pakistan lies in the locale of arid and semi-arid terrain characterized by severe climatic circumstances, with an average annual precipitation of 250–500 mm and temperatures ranging from 20 to 40 °C^9^. Consequently, drought stress poses a significant challenge to food security in this region^10^.

Among all cereal crops, wheat is the favorite food for human beings and as feed for animals too^11^. Wheat and its different byproducts in human diet is basically the inexpensive protein and human calories intake^12^. Estimatedly, 60% of the daily calorie intake is relied on this small grained crop and that’s why it is envisaged as the most important cereal crop in the world keeping in view its total production, cultivated area, and human consumptions^13^. So, it can be a very helpful source for efficient and energetic human health globally. In Pakistan, wheat planting is done under wide ranged soil profiles and climatic situations^14^. To sustain healthy plant life, water serves as a basic unit and its limited supply or unavailability may cause ultimate plant death. Wheat yield is severely affected by drought, particularly at critical growth stages like tillering and anthesis^15^. Drought directly upsets the grain formation in wheat^16,17^.

Previously, Hernández-Espinosa et al.^18^ experimented for two years under six environmental conditions with 54 genotypes of bread wheat and concluded; Grain morphology (specifically grain density and size), as well as Grain quality (specially protein, and flour quality) were severely affected by drought and heat stress. In the past, Ivanova et al.^19^ observed the effect of drought stress on the seven wheat varieties and reported that drought significantly affected the growth and yield of all tested wheat varieties. Furthermore, the aftermaths of their research results indicated that; 40%, 29%, 15%, and 30% reduction in grain weight, the grains per spike, grain size, and grain yield, respectively were recorded in drought-affected wheat plants as compared to the control treatment. Recently, Ojuederie et al.^20^ commenced that climate change has a link with global warming. Hence, indispensable innovative technologies are required for food security, especially in the developing world. To cope with drought-induced plant deaths, various strategies are being adopted now a days and among all those, the application of nanomaterials (NMs) has been proven as a promising and effective one. Consequently, various NMs have been reported to enhance crop production which is a go ahead to meet the growing global demands for food, feed, and fuel^1,4^.

Nanomaterials are natural, incidental, or manufactured materials containing particles, in; an unbound state, an aggregate, or as an agglomerate with size in the range of 1–100 nm^21^. Nanotech research and development in the agricultural crop sciences is likely the forecast of coming events regarding the next level expansion of genetically modified crops and precision farming techniques^22^; For the emerging world in agricultural prospects, the drive is to develop drought and pest-resistant crops^23^. Hence, it has many contributions to crop production as well as to eco-economic stability. The entire value chain of the agricultural production system can be subjugated with the help of nanoparticles^24^. Nanotechnology in agriculture will grow faster in near future with explanations of profound collaborations between nanoparticles (NPs) and plants^25^. The presence of NPs inside the plant tissues can restrain oxidative stress enzymes and trigger the plant defense system^26^. The size and concentration of NPs, their mode of application, plant species, and soil conditions may mold them to be a source of advantageous or adversarial effects on plant efficiency^27^. Usually, the NPs influence the plants by a two phasic dose-reaction “hormesis” with an up-dose inhibition and low-dose stimulation^28^.

The distinct physicochemical properties of NPs make them capable to increase plant metabolism^29^. NPs with dimensional characteristics like lesser physique and outsized surface area are perfect materials for use in plants, offering a significant way to release trace elements step by step in an exact manner, and consequently; NPs have established their place and roles in agriculture^30^. Zinc (Zn) is an essential constituent of various dynamic enzymes (such as; glutamate dehydrogenase-GDH, catalase-CAT, and superoxide dismutase-SOD), and also take part in chlorophyll and hormone synthesis^31^. Arough et al.^32^ indicated the positive effects of Zn application under salt stress. Dimkpa et al.^33^ reported that nano-ZnO improved grain yield and nutrient translocation in drought-stressed situations. Similarly, nano-ZnO may recruit speedy use mechanisms of seed food reservoirs, zinc uptake, and expression of antioxidants under drought. So, the use of nano-ZnO augmented; seed germination, sprout progression, yield, and WUE in rice, soybean, and sunflower crops^34–36^. Furthermore, Elshayb et al.^37^ and Abbas et al.^38^ have reported the beneficial effects of ZnO-NPs on the growth, yield, and grain quality of rice and wheat, respectively, under drought. Therefore, current study aimed; an evaluation of different doses of zinc-oxide nanoparticles (ZnO-NPs) on drought-stressed wheat seedlings at; (1) tillering stage (D_1_) and, (2) grain-filling stage (D_2_). We hypothesize that the use of ZnO-NPs will improve the growth, water relations, productivity, physiology and quality of wheat grains under the drought stress.

## Materials and methods

### Crop husbandry

The current pot experiment was carried out in the wire house of Department of Agronomy, The Islamia University of Bahawalpur, Pakistan (Latitude: 29°23 × 60.00″ N, Longitude: 71°40 × 59.99″ E, and height from sea level approximately 117 m). The experiment was conducted in completely randomized design (CRD) with factorial settings having three replications. The treatments were assigned randomly to experimental units (pots) within each replication to ensure unbiased results and to enhance the reliability of the findings. Four different dose levels of ZnO-NPs (ZnO-NPs_0_ = 0 ppm, ZnO-NPs_1_ = 50 ppm, ZnO-NPs_2_ = 100 ppm, and ZnO-NPs_3_ = 150 ppm) were evaluated against drought stress at critical growth stages (D_0_ = no drought, D_1_ = drought at tillering, D_2_ = drought at grain filling) of wheat. The seeds of wheat genotype (Galaxy 2013) were purchased from the Regional Agricultural Research Institute, Bahawalpur. The seeds were surface disinfected with 0.5% sodium hypochlorite (NaOCl) solution by dipping for 5 min and subsequently, washed many times by using distilled water. Ten seeds were planted on November 15, 2019, in each pot (30 × 30 cm) filled with 15 kg clay loam dry soil, amended with ZnO-NPs according to the study scheme, under wire house conditions with weather specifications of 70 ± 5% relative humidity and a day/night temperature of 31/20°C, respectively. The NPK was fertilized at a basal dose of 150:100:60 kg ha^− 1^ at sowing time. Nitrogen was split into 2 doses i.e., half with P, K, and the other half after 3 weeks of plant growth. Periodically, random rotation of pots was kept continuously and manual weeding was done regularly. The results of the physio-chemical analysis of soil samples are presented in Table 1. A transparent plastic sheet was used to cover the wire house to avoid rain, when required. After two weeks of germination, the plants were thinned, leaving only five uniform plants per pot.

**Table 1:** Initial physical and chemical characteristics of the experimental soil (0–20 cm depth).

Soil character type	Parameters	Units	Sample results
Soil physical properties	Sand	%	40
	Silt	%	42
	Clay	%	24
	Texture class	-	Clayey loam soil
Soil chemical properties	pH (1:2.5)	-	7.72
	CEC	cmol_c_/kg	3.34
	Electric conductivity (1:2.5)	dS m^− 1^	2.20
	Sodium absorption ratio	(mmol_c_/L)^1/2^	4.38
Soil mineral data (Macro and micro-nutrients)	Ammonical nitrogen (N)	mg g^− 1^	1.62
	Available phosphorus (P)	ppm	7.68
	Available potassium (K)	ppm	116
	Copper (Cu)	mg Kg^− 1^	16.75
	Manganese (Mn)	mg Kg^− 1^	54.67
	Zinc (Zn)	mg Kg^− 1^	37.87
	Iron (Fe)	mg Kg^− 1^	188.56
Soil organic data	Organic matter	%	0.90

(1:2.5) indicates soil / water ratio.

### Drought imposition and characterization of ZnO-NPs

All the pots were irrigated uniformly until the imposition of drought at tillering and grain filling stages. The 25% water holding capacity (WHC) was maintained in pots under drought at critical growth stages (tillering and grain filling) of wheat and 80% WHC was considered as control. The soil WHC was determined before the experiment by saturating the soil and measuring the water retained after drainage. The required amount of water was calculated based on pot weight, and soil moisture content were monitored regularly using a Theta probe (soil moisture sensor). Adjustments were made as needed by withholding or adding water to ensure that soil moisture content remained at the target WHC throughout the experiment.

**Fig. 1:**
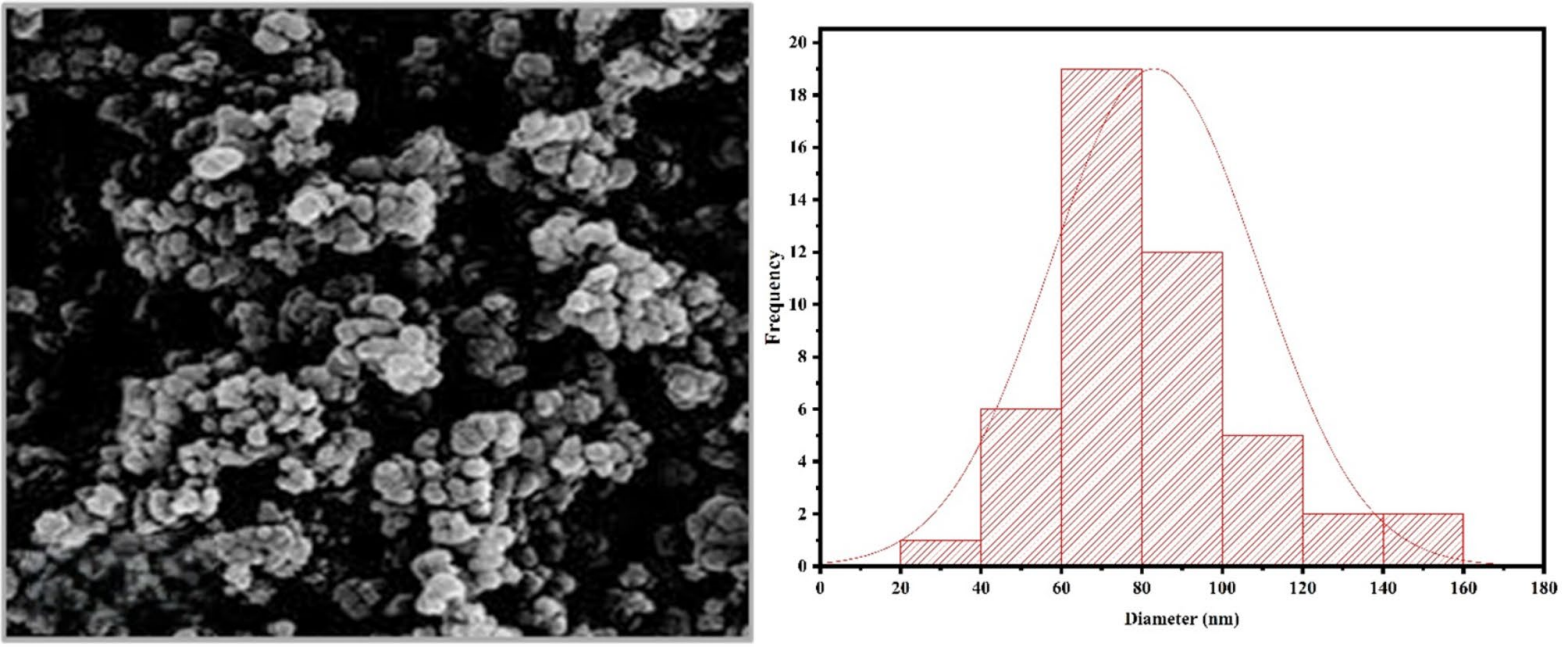
SEM-scanning image and average size distribution of ZnO nanoparticles (ZnO-NPs) used in the current study.

Zinc oxide nanoparticles (ZnO-NPs) used in the current study were sourced as zinc oxide nano powder (Assay ~ 80% zinc basis, MW 81.39 g/mol) from SIGMA ALDRICH^®^, USA. The ZnO-NPs were characterized to confirm their morphology, size, and purity. The X-ray diffraction spectroscopy (XRD) revealed 99% purity with an average particle size of 70 nm, 10–25 m^2^g^− 1^ surface area, and 5.606 gm^− 3^ density. The morphology and topology of ZnO-NPs were studied using Scanning Electron Microscope (SEM). The SEM analysis represented the spherical-shaped nanoparticles that were agglomerated as previously reported by Ismail et al.^39^ (Fig. 1).

### Plant harvesting and sampling procedures

The wheat plant samples were harvested at maturity stage after 18 weeks of sowing to measure growth, physiology, and yield-related attributes. Similarly, root and shoot samples were also taken to measure the length and dry weights. The harvested root samples were washed with 0.1 M HCl to remove any unwanted contaminations, followed by rewashing with distilled water. Samples were oven dried at 70 °C (Wiseven oven Model Won-155) for 72 h for root and shoot dry weights. Then, samples were crushed into small pieces for other physiological determinations.

### Measurements of leaf water relations

Following the techniques considered by, Thomas et al.^40^ for recording different measurements at different growth stages, the calculation of the Leaf area index (LAI) was done as the ratio of the surface area of a leaf to the ground surface where a plant stands.

$$\:\text{L}\text{A}\text{I}=\frac{\text{L}\text{e}\text{a}\text{f}\:\text{s}\text{u}\text{r}\text{f}\text{a}\text{c}\text{e}\:\text{a}\text{r}\text{e}\text{a}}{\text{G}\text{r}\text{o}\text{u}\text{n}\text{d}\:\text{s}\text{u}\text{r}\text{f}\text{a}\text{c}\text{e}\:\text{a}\text{r}\text{e}\text{a}\:\left(\text{P}\text{l}\text{a}\text{n}\text{t}\right)}$$

From the top of the wheat plants, the 3rd number leaf (fully expanded and the youngest one) of all ten plants in each treatment was taken to determine the excised leaf water loss (ELWL). Then, these leaves were placed in polythene bags and transported all together to the laboratory as quickly as possible to minimize water losses (due to evaporation). These sampled leaves were weighed at three stages, immediately after sampling (fresh weight) (1st stage), then placed the leaves at room temperature for 6 h and wilted weight was obtained (2nd stage), and then dried in an oven for 24 h at 70 °C and the dry weight was taken out (3rd stage). Moreover, excised leaf water loss was calculated by using the following formula as given by Clarke^41^.

$$\:\text{E}\text{L}\text{W}\text{L}\:\left(\text{\%}\right)=\:\frac{\text{F}\text{r}\text{e}\text{s}\text{h}\:\text{w}\text{e}\text{i}\text{g}\text{h}\text{t}-\text{W}\text{i}\text{l}\text{t}\text{e}\text{d}\:\text{w}\text{e}\text{i}\text{g}\text{h}\text{t}}{\text{D}\text{r}\text{y}\:\text{w}\text{e}\text{i}\text{g}\text{h}\text{t}}\:\text{X}\:100$$

Relative water contents of leaves were calculated as described by Barrs & Weatherley^42^. Briefly, the fresh weight of fully expended wheat leaves just collected from the field was measured immediately after being carried to the laboratory in plastic bags to avoid loss of water contents. A turgid weight of leaf samples (W_t_) was taken by soaking them for 16–18 h under normal conditions (room temperature), and then dried with tissue paper and weighed again. These leaves were oven dried for 72 h at 70 °C and were weighed to record dry weight (W_d_).

$$\:\text{R}\text{W}\text{C}=\:\frac{{\text{W}}_{\text{f}}-{\text{W}}_{\text{d}}}{{\text{W}}_{\text{t}}-{\text{W}}_{\text{d}}}\:\times\:100\:\:\:\:$$

W_f_ indicates fresh weight, W_d_ indicates dry weight, and W_t_ indicates turgor weight.

### Measurement of gas exchange and physiological traits

Leaf gas exchange parameters, such as stomatal conductance (m mol m^− 2^ s^− 1^), transpiration rate (µ mol m^− 1^ s^− 2^), and net photosynthetic rate (µ mol m^− 1^ s^− 1^), were measured using an infrared gas analyzer (Li-COR LI-6250, Li-COR Biosciences, Herts, England). The measurements were taken between 09:00 AM to 10:00 AM on clear, sunny days to lessen the diurnal variability. The topmost fully expanded and healthy young leaves were selected to ensure uniformity in sampling and to avoid bias due to leaf age or damage.

Chlorophyll contents were determined 80 days after seed sowing using fully expanded leaves. A 50 mg leaf sample was extracted in 5 ml of 80% acetone in dark at 4 °C for about 24 h to prevent chlorophyll degradation and the solution was then filtered. The absorbance of the filtered solution was measured with a spectrophotometer (Model; Halo DB-20/DB-20 S, f Dynamica Company, London, UK) at 470, 647 and 664.5 nm wavelengths. Then, the chlorophyll contents were calculated using the equations described by Lichtenthaler^43^.

### Measurement of yield and quality-related grain attributes

Agronomic attributes like tillering count per plant, plant height (cm), spike length (cm), root and shoot lengths (cm), number of grains per spike, test weight (1000 grain weight, g), biomass production per plant (g) and grain yield per plant (g) were calculated and recorded following the standard procedures and protocols. Plant height was measured by selecting five plants from each pot and measuring the height from the soil surface to the spikelet with the help of a measuring tape. A centimeter scale was used to measure spike length (from base to terminal spikelet) from seven random plants and counted the grains from these spikes too. The digital balance was used to quantify 1000-grain weight accurately.

The grain quality traits like protein and zinc concentrations were also tested. Colorimetrically estimated grain N content^44^ from 0.1 g of grain digested material was converted into protein content^45^. Additionally, ICP-OES (Model - Optima-2100–DV, Perkin-Elmer, Waltham, MA, USA) was used to determine zinc concentration from digested grain samples. The nutrients uptake by shoots i.e., N, P, and K were measured in consistence with Wolf^46^.

$$\:\text{N}\:\text{c}\text{o}\text{n}\text{t}\text{e}\text{n}\text{t}\:\times\:5.70=\text{P}\text{r}\text{o}\text{t}\text{e}\text{i}\text{n}\:\text{c}\text{o}\text{n}\text{t}\text{e}\text{n}\text{t}\:$$

### Statistical analysis

All the recorded parameters were analyzed using a statistical software (Statistix 8.1). The analysis of variance (ANOVA) was performed to check the significance of the treatment effects. When the significant differences were observed, the treatment means were compared using Fisher’s least significant difference (LSD) test at 5% probability level. Then, the data were also analyzed for the principal component analysis (PCA) using R software (2019) to check the association among different growth, yield, and quality attributes. The choice of these methods aligns with the objectives of the study to evaluate the treatment effects and interrelations among measured traits comprehensively (Tables 2 and 3).

**Table 2:** Effect of different doses of ZnO-NPs on yield contributing attributes of wheat under various stage-based drought levels.

Drought (D)	ZnO-NPs	Yield contributing traits						
		NSPS	NGS	Test Wt.	BP	GY	SL	TLP
D_0_ = no drought (control)	0 ppm	21.77 ± 0.39 ^**abcd**^	42.98 ± 0.20 ^**cd**^	32.96 ± 0.98 ^**b**^	8.05 ± 0.54 ^de^	1.19 ± 0.02 ^**c**^	11.76 ± 2.11 ^**ab**^	4.63 ± 0.24 ^**ab**^
	50 ppm	21.82 ± 1.08 ^**abc**^	43.10 ± 0.96 ^**c**^	33.18 ± 0.02 ^**b**^	8.08 ± 0.96 ^**d**^	1.26 ± 0.06 ^**b**^	12.00 ± 1.36 ^**ab**^	4.65 ± 0.23 ^**ab**^
	100 ppm	22.26 ± 0.46 ^**ab**^	43.96 ± 1.05 ^**bc**^	33.97 ± 0.30 ^**b**^	8.78 ± 1.09 ^**b**^	1.29 ± 0.09 ^**b**^	12.27 ± 1.21 ^**a**^	4.82 ± 0.81 ^**ab**^
	150 ppm	22.74 ± 1.08 ^**a**^	45.92 ± 0.6 ^**a**^	35.56 ± 1.00 ^**a**^	10.98 ± 0.07 ^**a**^	1.38 ± 0.23 ^**a**^	13.23 ± 2.31 ^**a**^	6.12 ± 0.31 ^**a**^
D_1_ = drought at tillering	0 ppm	20.40 ± 2.44 ^**cde**^	40.00 ± 2.00 ^**f**^	29.62 ± 1.94 ^**c**^	7.04 ± 0.45 ^**fg**^	1.02 ± 0.14 ^**d**^	10.52 ± 0.36 ^**bc**^	3.00 ± 0.10 ^**c**^
	50 ppm	20.69 ± 1.04 ^**cde**^	40.54 ± 0.76 ^**ef**^	29.92 ± 1.49 ^**c**^	7.06 ± 0.65 ^**fg**^	1.16 ± 0.09 ^**c**^	11.84 ± 1.51 ^**ab**^	3.00 ± 0.27 ^**c**^
	100 ppm	20.98 ± 1.00 ^a**bcde**^	41.78 ± 0.87 ^**de**^	30.21 ± 1.06 ^**c**^	7.89 ± 1.04 ^**e**^	1.19 ± 0.06 ^**c**^	12.00 ± 1.87 ^**ab**^	3.24 ± 0.78 ^**bc**^
	150 ppm	21.79 ± 0.60 ^**abcd**^	44.98 ± 0.06 ^**ab**^	33.32 ± 0.86 ^**b**^	8.58 ± 0.26 ^**c**^	1.28 ± 0.14 ^**b**^	12.92 ± 2.21 ^**a**^	4.56 ± 0.69 ^**abc**^
D_2_ = drought at grain filling	0 ppm	19.82 2.00 ± ^**e**^	36.46 ± 1.23 ^**h**^	26.12 ± 2.10 ^**d**^	6.24 ± 0.02^**i**^	0.96 ± 0.08 ^**e**^	9.87 ± 1.14 ^**c**^	3.00 ± 0.42 ^**c**^
	50 ppm	20.00 ± 1.00 ^**de**^	37.24 ± 0.98 ^**gh**^	26.56 ± 0.6 ^**d**^	6.55 ± 1.42 ^**h**^	0.98 ± 0.05 ^**de**^	10.00 ± 1.04 ^**c**^	3.00 ± 0.07 ^**c**^
	100 ppm	20.64 ± 1.97 ^**bcde**^	38.50 ± 0.95 ^**g**^	26.88 ± 2.18 ^**d**^	6.96 ± 0.43 ^**g**^	1.03 ± 0.08 ^**d**^	10.68 ± 1.13 ^**bc**^	3.00 ± 0.76 ^**c**^
	150 ppm	21.90 ± 1.03 ^**abc**^	40.52 ± 0.64 ^**ef**^	27.42 ± 0.65 ^**d**^	7.21 ± 2.10 ^**f**^	1.15 ± 0.04 ^**c**^	11.85 ± 0.51 ^**bc**^	4.00 ± 0.20 ^**bc**^
**LSD**_**0.05**_	D×ZnO-NPs	1.79	1.29	1.35	0.19	0.06	1.52	1.62
**Std. Error**	D×ZnO-NPs	0.86	0.63	0.65	0.09	0.03	0.74	0.78

The different lower-case letters (a, b, c, d) exhibited in each column represent statistically significant differences among the studied treatments through LSD test at 5% probability index (*P* ≤ 0.05). The abbreviations are as following: ZnO-NPs = Zinc oxide nanoparticles, NSPS = No. of spikelets per spike, NGS = No. of grains per spike, Test Wt. = 1000 grain weight (g), BP = Biomass production (g), GY = grain yield / plant (g), TLP = Number of tillers per plant, and SL = Spike length (cm).

**Table 3:** Effect of different doses of ZnO-NPs on leaf gas exchange and grain quality traits of wheat under various stage-based drought levels.

Drought (D)	ZnO-NPs	Plant height, gas exchange and grain quality traits					
		PH	SC	Tr	Pn	Gzn	Gprot.
D_0_ = no drought (control)	0 ppm	94.00 ± 5.16 ^**bcd**^	437.70 ± 0.17 ^**a**^	1.82 ± 0.17 ^**a**^	15.87 ± 1.10 ^**a**^	11.98 ± 0.22 ^**de**^	9.00 ± 1.00 ^**f**^
	50 ppm	95.00 ± 3.73 ^**bc**^	422.21 ± 0.43 ^**d**^	1.84 ± 0.04 ^**a**^	15.87 ± 0.04 ^**a**^	12.00 ± 2.00 ^**de**^	9.25 ± 0.25 ^**ef**^
	100 ppm	96.00 ± 2.97 ^**ab**^	427.43 ± 0.21 ^**c**^	1.86 ± 0.10 ^**a**^	15.89 ± 0.00 ^**a**^	13.25 ± 0.34 ^**cd**^	9.65 ± 0.05 ^**e**^
	150 ppm	98.00 ± 7.04 ^**a**^	432.36 ± 0.39 ^**b**^	1.90 ± 0.06 ^**a**^	15.94 ± 0.01 ^**a**^	14.76 ± 0.10 ^**c**^	10.42 ± 0.02 ^**d**^
D_1_ = drought at tillering	0 ppm	89.00 ± 6.61 ^**g**^	423.03 ± 1.03 ^**d**^	0.41 ± 0.04 ^**d**^	5.95 ± 0.00 ^**d**^	11.76 ± 0.26 ^d**e**^	11.60 ± 0.36 ^**c**^
	50 ppm	90.40 ± 6.05 ^**fg**^	408.00 ± 2.00 ^**f**^	0.49 ± 0.01 ^**d**^	6.03 ± 1.15 ^**d**^	15.00 ± 1.01 ^**c**^	11.68 ± 0.27 ^**c**^
	100 ppm	91.00 ± 2.96 ^**efg**^	412.76 ± 1.12 ^**e**^	0.61 ± 0.01 ^**c**^	7.94 ± 0.08 ^**c**^	18.00 ± 2.00 ^**b**^	11.76 ± 0.08 ^**c**^
	150 ppm	92.00 ± 6.11 ^**def**^	413.05 ± 0.02 ^**e**^	0.91 ± 0.17 ^**b**^	9.94 ± 1.00 ^**b**^	19.00 ± 2.16 ^**b**^	11.84 ± 0.20 ^**c**^
D_2_ = drought at grain filling	0 ppm	93.00 ± 5.88 ^**cde**^	413.05 ± 1.00 ^**e**^	0.21 ± 0.14 ^**e**^	3.95 ± 0.00 ^**e**^	9.87 ± 0.07 ^**e**^	12.85 ± 1.01 ^**b**^
	50 ppm	94.25 ± 4.76 ^**bcd**^	398.00 ± 2.21 ^**h**^	0.26 ± 0.05 ^**e**^	3.98 ± 1.00 ^**e**^	12.00 ± 2.11 ^**de**^	12.92 ± 0.00 ^**b**^
	100 ppm	94.87 ± 5.49 ^**bc**^	402.78 ± 1.23 ^**g**^	0.41 ± 0.03 ^**d**^	5.93 ± 0.86 ^**d**^	18.13 ± 0.03 ^b^	12.99 ± 0.01 ^**b**^
	150 ppm	96.00 ± 6.05 ^**ab**^	407.71 ± 3.00 ^**f**^	0.41 ± 0.08 ^**d**^	5.94 ± 0.00 ^**d**^	24.00 ± 2.00 ^**a**^	13.84 ± 0.06 ^**a**^
**LSD**_**0.05**_	D×ZnO-NPs	2.44	2.32	0.11	0.49	2.32	0.51
**Std. Error**	D×ZnO-NPs	1.19	1.12	0.05	0.23	1.12	0.24

Means sharing the same letters are not significantly different at a 5% probability level. The abbreviations are as following: ZnO-NPs = Zinc oxide nanoparticles, PH = Plant height (cm), SC = Stomatal conductance (m mol m^− 2^ s^− 1^), Tr = Transpiration rate (µ mol m^− 1^ s^− 2^), Pn = Photosynthetic rate (µ mol m^− 1^ s^− 1^), Gzn = Grain zinc conc. (mg kg^− 1^ DW), and Gprot. = Grain protein (%).

## Results

### Effects of various doses of ZnO-NPs on growth attributes of wheat under drought

**Fig. 2:**
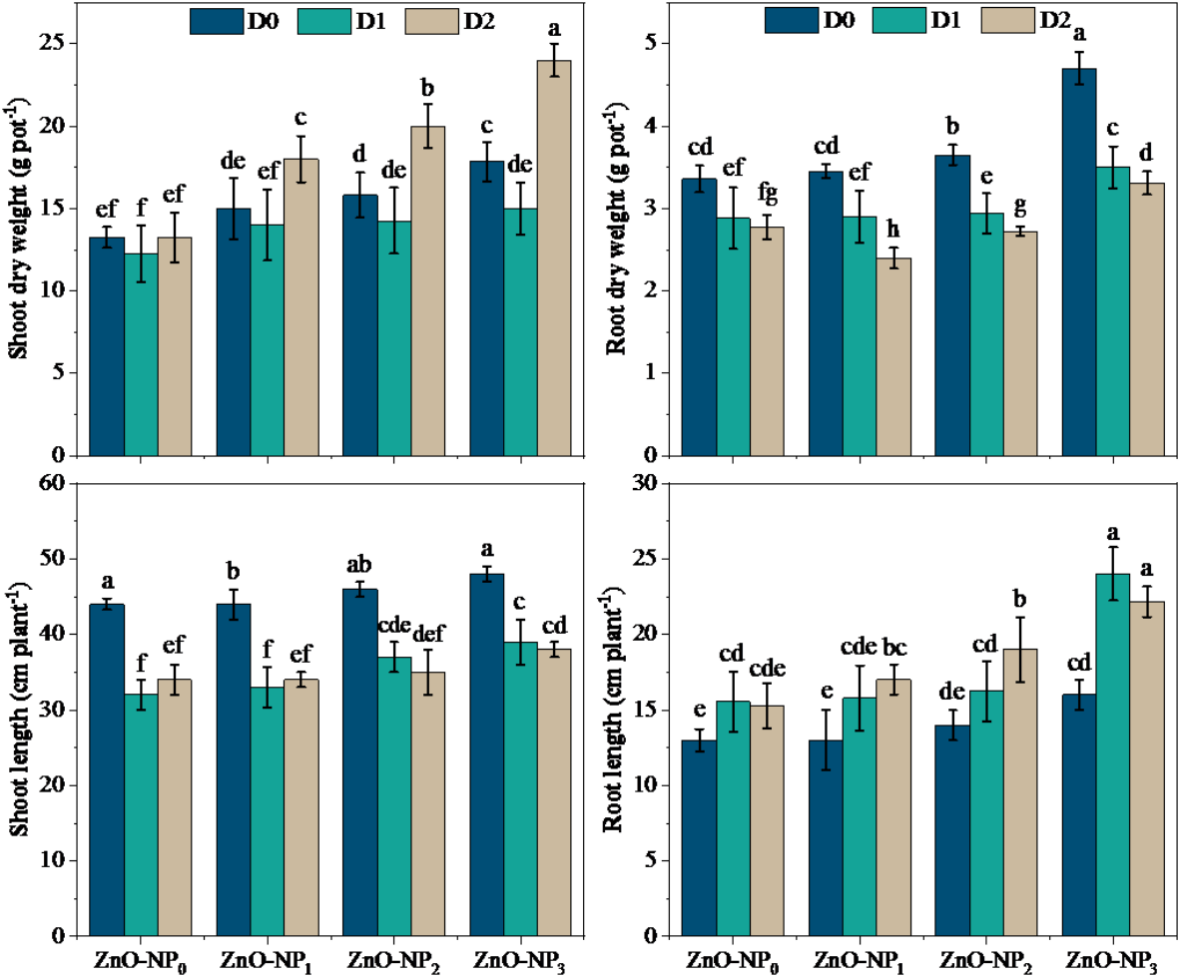
Effect of different doses of zinc oxide nanoparticles on morphological traits of wheat under various stage-based drought levels. The data presented in the form of each bar is an average of three replications (*n* = 3). The different lower-case letters (a, b, c, d) exhibited on the bars represent statistically significant differences among the studied treatments through LSD test at 5% probability index (*P* ≤ 0.05). The abbreviations are as following: D_0_ = no drought (control), D_1_ = drought at tillering, D_2_ = drought at grain filling, ZnO-NP_0_ = zinc oxide nanoparticles at 0 ppm, ZnO-NP_1_ = zinc oxide nanoparticles at 50 ppm, ZnO-NP_2_ = zinc oxide nanoparticles at 100 ppm, and ZnO-NP_3_ = zinc oxide nanoparticles at 150 ppm.

Various parameters like plant height, root length, shoot length, and root-shoot dry weight under drought stress at tillering (D_1_) and grain filling (D_2_) stages demonstrated significant improvements upon application of different doses of ZnO-NPs over control (Table 4). During the current study, shoot and root dry weight followed an increasing trend under the application of ZnO-NPs over drought or un-droughted treatments without ZnO-NPs applications. Overall, the application of ZnO-NPs (150 ppm) at D_2_ produced more shoot dry weight followed by 100 and 50 ppm NPs concentrations. Briefly, supply of 150 ppm (ZnO-NPs_3_) at the D_2_ drought level amplified the shoot dry weight by 81.2%, as compared to D_0_×ZnO-NPs_0_ treatment. Similarly, the application of ZnO-NPs_3_ level exhibited the maximum increase in root dry weight (39.9%), followed by ZnO-NPs_2_ (8.6%), and ZnO-NPs_1_ (4.2%), as compared with the D_0_×ZnO-NPs_0_ treatment (Fig. 2). A significant reduction in shoot length was shown by D_1_, while drought at both D_1_ and D_2_ didn’t affect root length, as compared with control (D_0_). The data revealed taht application of ZnO-NPs increased plant height, shoot and root length under drought and control treatments. The ZnO-NPs under D_1_ increased shoot length by 3.1%, 15.6%, and 21.9% at 50, 100, and 150 ppm concentration respectively as compared with shoot length under D_1_ without ZnO-NPs application (0 ppm). On the other hand, D_2_ treatment showed a maximum shoot length increment of 11.8% at 150 ppm NPs (ZnO-NPs_3_) as compared to similar D_2_ drought level with 0 ppm ZnO-NPs application rate (Fig. 2; Table 3).

**Table 4:** Summary of the ANOVA for plant height (PH, cm), leaf area index (LAI, ), root length (RL, cm), shoot length (SHL, cm), root dry weight (RDW, g), shoot dry weight (SDW, g), number of tillers per plant (TLP), spike length (SL, cm), number of spikelets per spike (NSPS), number of grains per spike (NGS), 1000 grain weight (test Wt., g), biomass production (BP, g), grain yield / plant (GY, g).

SOV	PH	LAI	RL	SHL	RDW	SDW	TLP	SL	NSPS	NGS	Test wt.	BP	GY
Drought (D)	**	**	**	**	**	**	**	**	**	**	**	**	**
ZnO-NPs	**	**	**	**	**	**	**	**	**	**	**	**	**
D×ZnO-NPs	NS	**	*	NS	**	**	NS	NS	NS	NS	NS	**	*

Significant differences are indicated by an (*); *P ≤ 0.05, **P ≤ 0.001, NS, Non-significant, SOV, sources of variation.

### Effects of various doses of ZnO-NPs on yield-related traits of wheat under drought

The data regarding different yield-related attributes of drought-stressed wheat under the effect of ZnO-NPs is given in Table 2. It was observed that drought stress on grain filling stage (D_2_) with no NPs amendment more negatively affected yield related attributes as compared to drought at tillering stage (D_1_). However, ZnO-NPs application significantly alleviated the toxic impacts of drought at both growth stages (tillering and grain filling) and improved grain yield and its related traits in wheat crop. Particularly, at D_0_ drought stress, the findings showed that ZnO-NPs_3_ (150 ppm) application level increased No. of grains per spike (6.8%), 1000 grain weight (7.9%), grain yield per plant (16.0%), no. of tillers (32.2%), and spike length (12.5%) followed by ZnO-NPs_2_ (100 ppm) and ZnO-NPs_1_ (50 ppm), as compared with D_0_×ZnO-NPs_0_ interaction. While at D_2_ drought stress, application of ZnO-NPs_3_ (150 ppm) prominently enhanced the no. of grains per spike, 1000 grain weight, grain yield per plant, no. of tillers per plant, and spike length by 11.2%, 5.1%, 19.8%, 33.3%, and 20.1% correspondingly, as compared with the D_2_×ZnO-NPs_0_ treatment (Table 4).

### Effects of various doses of ZnO-NPs on physiology of wheat under drought

**Fig. 3:**
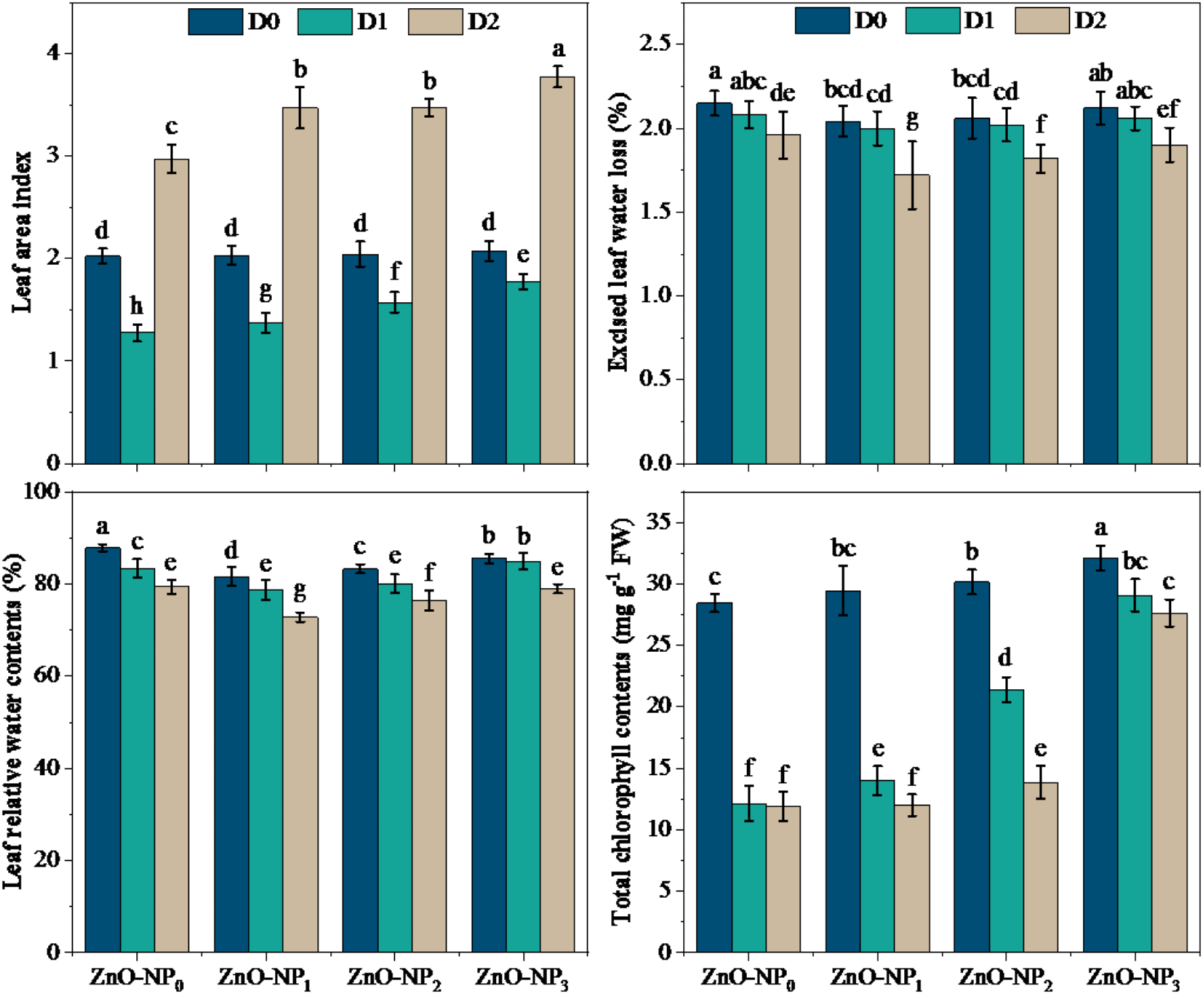
Effect of different doses of ZnO-NPs on leaf water relations of wheat under various stage-based drought levels. The different lower-case letters (a, b, c, d) exhibited on the bars represent statistically significant differences among the studied treatments through LSD test at 5% probability index (*P* ≤ 0.05). The data presented in the form of each bar is an average of three replications (*n* = 3). The abbreviations are as following: D_0_ = no drought (control), D_1_ = drought at tillering, D_2_ = drought at grain filling, ZnO-NP_0_ = zinc oxide nanoparticles at 0 ppm, ZnO-NP_1_ = zinc oxide nanoparticles at 50 ppm, ZnO-NP_2_ = zinc oxide nanoparticles at 100 ppm, ZnO-NP_3_ = zinc oxide nanoparticles at 150 ppm.

The findings presented in Fig. 3 describe the adverse impacts of drought on different **leaf-related** parameters like leaf area index (LAI), excised leaf water loss (ELWL, %), leaf relative water content (LRWC, %), and chlorophyll contents. All the parameters, excluding LAI, followed a declining trend across drought treatments even when ZnO-NPs, when applied at lower doses. Interaction of drought and ZnO-NPs represented the highest ELWL and LRWC at D_0_×ZnO-NPs_0_ followed by D_0_×ZnO-NPs_3_, D_1_×ZnO-NPs_0,_ and D_1_×ZnO-NPs_3_ respectively. It was noticed that drought stress at tillering stage (D_1_) showed the lowest LAI after the application of lesser doses of ZnO-NPs (50 and 100 ppm), compared with the LAI at grain filling stage drought treatment (D_2_). For example, after application of ZnO-NPs_3_ (150 ppm rate) at D_2_ drought treatments exhibited an increment of 45.0% in LAI, over relevant ZnO-NPs_3_ treatment without drought stress (D_0_). Meanwhile, drought stress at tillering (D_1_) and grain filling (D_2_) stages showed a significant decrease in LRWC by 5.1% and 9.6%, while a significant increase was shown in chlorophyll contents by 12.90% and 2.21%, respectively, over un-droughted treatment without any NPs dose (D_0_×ZnO-NPs_0_). At the other end, the dose level of 150 ppm NPs (ZnO-NPs_3_) significantly increased LRWC at tillering stage under drought stress (D_1_), but no dose level of ZnO-NPs at grain filling could perform any beneficial effect. For example, D_1_ drought stress, the findings showed that ZnO-NPs_3_ (150 ppm) application level increased leaf relative water contents (LRWC) by 3.5%, and chlorophyll contents by 139.4% followed by ZnO-NPs_2_ (100 ppm), as compared with D_1_×ZnO-NPs_0_ interactive treatment (Tables 4 and 5).

**Table 5:** Summary of the ANOVA for chlorophyll (Chl., mg g^− 1^ fresh weight), leaf relative water content (LRWC, %), excised leaf water loss (ELWL, %), stomatal conductance (SC, m mol m^− 2^ s^− 1^), transpiration rate (tr, µ mol m^− 1^ s^− 2^), photosynthetic rate (pn, µ mol m^− 1^ s^− 1^), grain zinc conc. (Gzn, mg kg^− 1^ DW), grain protein (Gprot., %), Nitrogen, phosphorous and potassium uptakes (N, P, K, mg g^− 1^ fresh weight).

SOV	Chl.	LRWC	ELWL	SC	Tr	Pn	Gzn	Gprot.	*N*	*P*	K
Drought (D)	**	**	**	**	**	**	**	**	**	**	**
ZnO-NPs	**	**	**	**	**	**	**	**	**	**	**
D×ZnO-NPs	**	**	NS	*	**	**	**	NS	*	*	**

Significant differences are indicated by an (*); *P ≤ 0.05, **P ≤ 0.001, NS, Non-significant, SOV, sources of variation.

### Effects of various doses of ZnO-NPs on leaf gas exchange of wheat under drought

A dissimilar crux in gaseous exchange attributes of wheat leaves i.e., leaf stomatal conductance (SC), transpiration rate (Tr), net photosynthetic rate (Pn) under the interactive effect of ZnO-NPs and drought conditions has been presented in Table 3. A significant difference among gas exchange traits under various drought levels was greatly o assessed. A great reduction was noticed in SC by 3.5% and 5.7%, Tr by 77.5% and 88.5%, and Pn by 62.6% and 75.4%, respectively, after the exposure of wheat seedlings with water stress at tillering (D_1_) and grain filling (D_1_) stages than un-droughted (D_0_) treatment. Nonetheless, the application of ZnO-NPs_2_ (100 ppm) and ZnO-NPs_3_ (150 ppm) treatments performed relatively better under drought and non-drought conditions compared with ZnO-NPs_1_ (50 ppm) treatment. For instance, an increment of 121.2% and 94.1% in Tr, and 67.3% and 51.1% in Pn rate was noticed under D_1_×ZnO-NPs_3_ and D_2_×ZnO-NPs_3_ treatments, accordingly, when compared with the relevant drought treatments without ZnO-NPs application. However, the effect of all ZnO-NPs doses was not significant on SC rate, in comparison with relevant control treatments (Table 5).

### Effect of different doses of ZnO-NPs on wheat grain quality under drought

The results regarding wheat grain quality indices such as grain zinc (Gzn) and protein contents (Gprot.) under the inter-active effect of ZnO-NPs and drought conditions has been presented in Table 3. The gain Zn contents were significantly reduced under the drought conditions at D_1_ and D_2_ stages without any dose of ZnO-NPs, and the highest decline of 17.6% in Gzn contents was noticed at D_2_×ZnO-NPs_0_ treatment (Table 5). However, supply of ZnO-NPs showed a worth-noticing increase in grain Zn contents under drought and non-drought growth conditions. For example, drought at tillering (D_1_) boosted Gzn contents by 62.1% and 53.1% under the use of ZnO-NPs_3_ (150 ppm) and ZnO-NPs_2_ (100 ppm), respectively, as compared with the same drought level without NPs amendment. On the other hand, significantly lowest grain protein contents were observed under all non-drought conditions. However, the drought stress alone or in combination with ZnO-NPs amendment showed a significantly positive impact on protein contents in grain, as compared to non-drought treatments. It was interesting to observe that grain protein accumulation efficiency was much higher at D_2_ drought level than the D_1_ drought level. The accumulation of grain protein contents was increased at both D_1_ and D_2_ drought stages under the application of ZnO-NPs_3_ by 3.5% and 8.1%, and ZnO-NPs_2_ by 3.1% and 2.1%, in comparison with the corresponding drought stage without ZnO-NPs, respectively.

### Effects of various doses of ZnO-NPs on nutrient uptake by wheat under drought

**Fig. 4:**
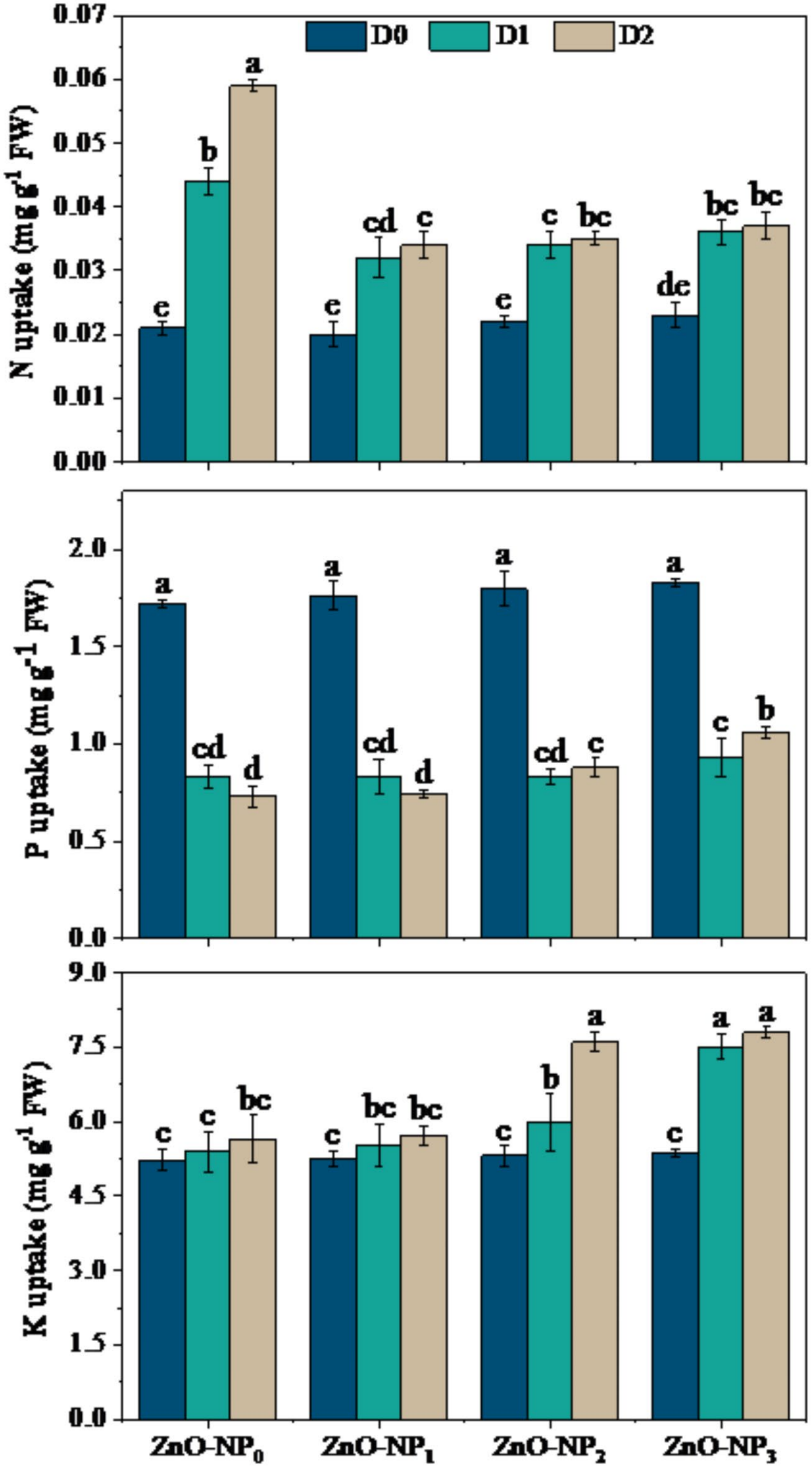
Effect of different doses of ZnO-NPs on nutrients (NPK) uptake by wheat leaves under various stage-based drought levels. The different lower-case letters (a, b, c, d) exhibited on the bars represent statistically significant differences among the studied treatments through LSD test at 5% probability index (*P* ≤ 0.05). The data presented in the form of each bar is an average of three replications (*n* = 3). The abbreviations are as following: D_0_ = no drought (control), D_1_ = drought at tillering, D_2_ = drought at grain filling, ZnO-NP_0_ = zinc oxide nanoparticles at 0 ppm, ZnO-NP_1_ = zinc oxide nanoparticles at 50 ppm, ZnO-NP_2_ = zinc oxide nanoparticles at 100 ppm, and ZnO-NP_3_ = zinc oxide nanoparticles at 150 ppm.

The data regarding the uptake of major nutrients by wheat crop e.g., nitrogen (N), phosphorous (P), and potassium (K) under the inter-active influence of ZnO-NPs and drought conditions has been presented in Fig. 4. In brief, the obtained findings suggested that N, and K showed a significant increase under drought-stressed treatments alone or in combination with ZnO-NPs dose levels. For example, the uptake of N was extensively increased under D_1_×ZnO-NPs_0_ by 109.5% and under D_2_×ZnO-NPs_0_ by 180.9%, when compared with the non-droughted treatment with no ZnO-NPs application (D_0_×ZnO-NPs_0_). Similarly, further increase in N (59.0% and 60.9%) and K (43.3% and 45.1%) uptake by wheat leaves was observed under the supply of ZnO-NPs_2_ and ZnO-NPs_3_ at grain filling drought stage (D_2_), accordingly, as compared with the D_0_ treatment in combination with the corresponding ZnO-NPs application rate. The response of phosphorous (P) uptake under various levels of ZnO-NPs and drought at tillering and grain filling stages was quite different than N and K uptake. There was no significant difference among the P uptake rates of wheat crop under all application rates of ZnO-NPs at D_0_ drought level. However, P uptake was significantly reduced by 53.5% and 48.7% under drought at tillering (D_1_), and by 51.3% and 42.1% under drought at grain filling stage (D_1_) after the application of ZnO-NPs_2_ and ZnO-NPs_3_ dose levels, as compared with same application rates of ZnO-NPs at D_0_ drought level.

### Principal components analysis (PCA)

**Fig. 5:**
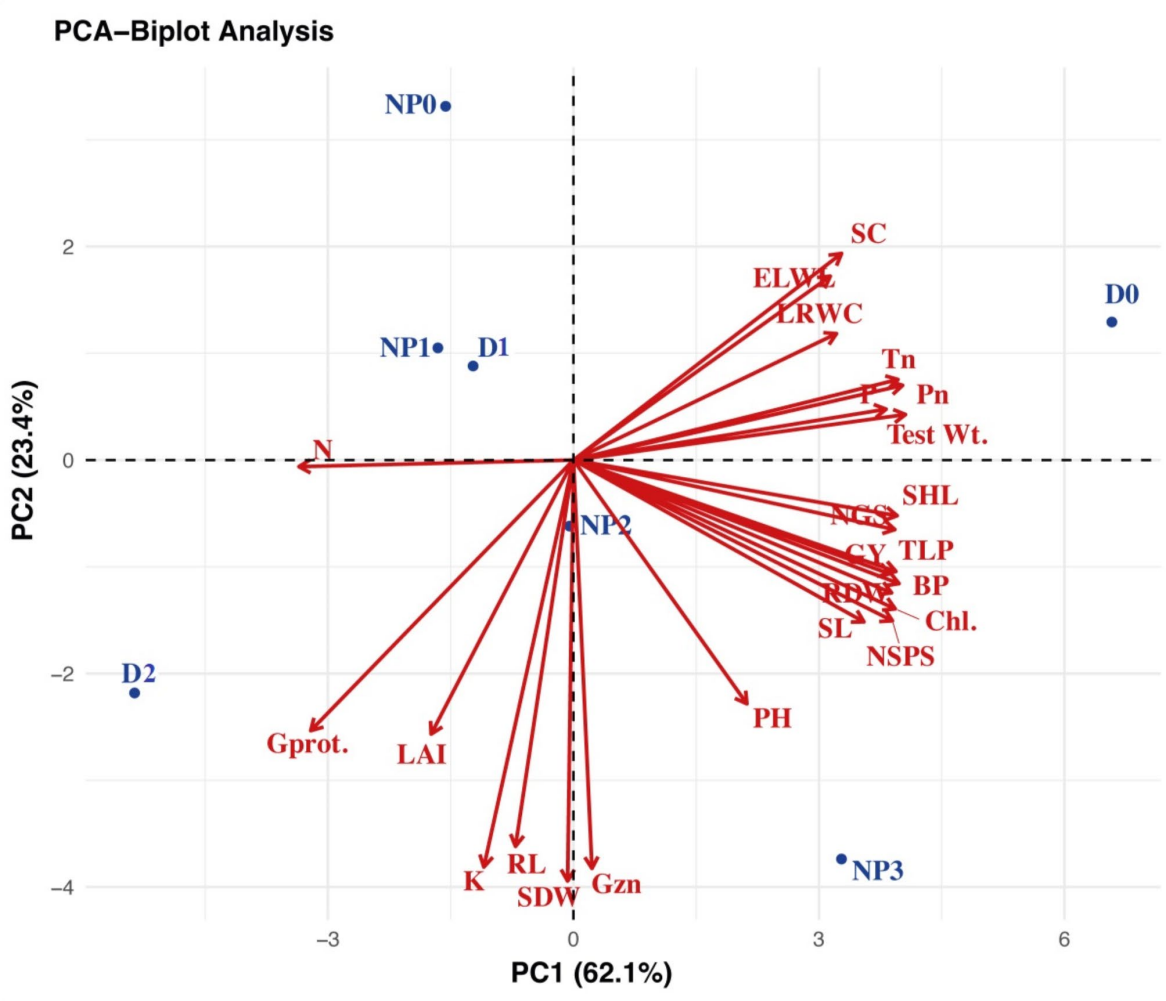
Principal component analysis (PCA) based on growth, physiological, yield, and quality parameters of wheat under different levels of ZnO-NPs and stage-based drought stress. The abbreviations are as following: D_0_ = no drought (control), D_**1**_ = drought at tillering, D_2_ = drought at grain filling, NP_0_ = zinc oxide nanoparticles at 0 ppm, NP_1_ = zinc oxide nanoparticles at 50 ppm, NP_2_ = zinc oxide nanoparticles at 100 ppm, NP_3_ = zinc oxide nanoparticles at 150 ppm, PH = plant height, LAI = Leaf area index, SL = spike length, NGS = number of grains per spike, Test Wt. = 1000 grain weight, GY = grain yield per plant (g), BP = Biomass production per plant (g), TLP = Tillers per plant, RL = Root length, SHL = Shoot length, RDW = Root dry weight, SDW = Shoot dry weight, Chl. = Chlorophyll, Pn = Photosynthesis rate, Tr = Transpiration rate, SC = Stomatal conductance, LRWC = Leaf relative water content, ELWL = Excised leaf water loss, N, P, K = Nitrogen, phosphorous and potassium uptakes, Gprot. = Grain protein, and Gzn = Grain zinc content.

To study the variations and associations among different growth, physiological, yield, and quality-linked parameters of wheat under the interaction of different; drought-stressed growth stages and dose levels of ZnO-NPs, the results were analyzed by performing a principal component analysis (PCA) to distinguish the beneficial treatments (Fig. 5). The treatments with different impacts on different parameters were scattered into different clades in PCA – Biplot Analysis (Fig. 5). The first two PCs (PC1 62.1% and PC2 23.4%) covered the 85.5% variability of the total variation from the 24 parameters. The first principal component (PC1) was related to all the parameters under study except nitrogen and K uptake (N, K), grain protein (Gprot.), leaf area index (LAI), root length (RL), and shoot dry weight (SDW) which were attributed to PC2. All the treatments of ZnO-NPs (0, 50, 100, and 150 ppm) under various drought intensities (D_0_ = Control, D_1_ = Tillering stage drought, and D_2_ = Grain filling stage drought) in wheat revealed variability among the treatments and stage-based drought episodes. Moreover, the amount of N and K uptake, grain protein, LAI, root length, and shoot dry weights were boosted under drought with negative eigenvalues.

## Discussion

Drought adversely affects the growth, yield and overall productivity of wheat, especially at critical growth stages^47,48^. The management of adverse drought impacts is possible by applying various strategies, including the use of nanotechnology^49^. Zinc oxide nanoparticles (ZnO-NPs) have shown potential in improving crop growth, grain yield and nutritional quality by promoting penological development in cereals^50^.

The shoots and roots are fundamental plant parts, with roots playing critical role in absorbing minerals and water from the soil, while also serving as sinks for certain nutrients. Our findings indicated that ZnO-NPs application effectively mitigated the ill effects of drought by enhancing both root and shoot lengths. This aligns with the previous studies by Singh et al.^51^, who reported increase root and shoot growth by the application of ZnO-NPs. Similarly, Sabir et al.^52^ and Solanki and Laura^53^ reported the significant increments in shoot and root lengths of *Zea mays* L. and wheat, respectively. Further, our study found decreased dry weights trends under drought over control but different doses of ZnO-NPs improved the root and shoot dry weights at tillering and grain filling stages under drought. So, ZnO-NPs have the potential to mitigate oxidative stress effects. Rizwan et al.^54^ also reported similar results by ZnO-NPs application on wheat and noticed a significant increase in root and shoot dry biomass under heavy metal stress might be due to the improved the nutrient uptake facilitated by ZnO-NPs.

Significant reduction in leaf area index (LAI) and leaf chlorophyll contents under drought stress conditions was due to the increased production of reactive oxygen species that triggers lipid peroxidation and subsequently chlorophyll destruction^55^. Similar outcomes have been reported by Aslam et al.^56^ in quinoa under drought conditions. ZnO-NPs (150ppm) enhance the cytokinin’s productions that improve metabolic activity and cell growth that significantly increase the LAI and chlorophyll. Likewise, lower concentrations of ZnO-NPs increased the LAI and chlorophyll content, whereas higher concentrations reduced these parameters in wheat subjected to drought conditions, as reported by Abbas et al.^38^. This reduction at higher concentrations is attributed to the negative effects of nanoparticles, which reduced the photosynthetic performance and antioxidant enzyme activities, ultimately leading to a decline in LAI and chlorophyll content^38^.

Drought stress significantly reduced the excised leaf water loss (ELWL) and leaf relative water content (LRWC) at tillering and grain filling stages as compared to the droughty control. Similarly, Elshayb et al.^37^ and Muhammad et al.^57^ reported a decrease in ELWL and LRWC under drought in rice and wheat, respectively, might be due to decreased cell division, soil moisture availability, and reduced plant ability to absorb nutrients. ZnO-NPs application enhanced the water relations (LRWC, ELWL) under both control and water-deficit conditions. Our findings align with previous studies, which reported that nanoparticles enhance the aforementioned parameters by increasing root formation and facilitating hormonal signaling, thereby increasing water uptake and maintaining better water relations under drought stress^33,37^.

Stomatal conductance (SC), transpiration rate (Tr), net photosynthetic rate (Pn) are important parameters to lookout the drought resilience in wheat. Drought imposed at tillering and grain filling stages reduced the soil water uptake, which eventually leads to closure of stomata and hence decreased the SC, Tr, and Pn as described in this study^58^. However, ZnO-NPs (150ppm) improved the SC, Tr, and Pn under both control and drought conditions. Likewise, Ahmed et al.^59^ and Seleiman et al.^60^ reported that ZnO-NPs improved the SC, Tr, and Pn in *Coriandrum sativum* L. and maize under drought and salt stress, respectively. This increase may result from improved water uptake, higher photosynthetic efficiency, enhanced phytohormones production and osmolyte accumulation, offering better resistance to wheat^59,61^.

The ZnO-NPs were reported to induce drought related tolerance in soybean (*Glycine max* L.) as reported by Sedghi et al.^34^. The number of tillers per plant in our study decreased more significantly under drought stress at tillering than at the grain filling stage. However, the highest dose of ZnO-NPs increased their number up to 52% more than the control treatment. A similar increase in the number of tillers in rice under arsenic stress was reported by the silica NPs^62^. Similarly, it has been reported that ZnO-NPs improved the number of tillers in wheat under drought conditions due to enhanced water and nutrient uptake^57^ .

Plant height (PH) is correlated with total biomass accumulation in plants. Drought negatively affected PH at tillering stage. Poor nutrient availability under drought stress could affect PH and spike length (SL) of wheat^63^. ZnO-NPs effectively increased PH and SL at each drought level. Raliya et al.^64^ reported increased PH in tomato plants by the application of ZnO-NPs (250 ppm). Similarly, an increase in PH and SL of wheat under drought stress has been reported by the application of ZnO-NPs in low concentrations, however, higher concentration (160 ppm) decreased the studied parameters due to toxicity, oxidative stress, and interference with nutrient uptake^38^.

Drought stress significantly reduced the grain protein and zinc content, uptake of nitrogen (N), phosphorus (P), and potassium (K) as compared to control treatment. Similarly, significant reduction in above mentioned parameters have been reported by Aslam et al.^56^ and Abbas et al.^38^ in quinoa and wheat, respectively, as drought stress reduced the uptake and translocation of nutrients from roots to aerials parts of plants due to limited water availability. However, ZnO-NPs (150ppm) improved the nutrient absorption and so increased the protein, zinc, N, P, and K in wheat grains as compared to control treatment. Similar results have been reported in various crops, demonstrating that ZnO-NPs enhance theses quality parameters, thereby supporting our findings.

The number of spikelets per spike (NSPS), number of grains per spike (NGS), 1000 grain weight, biomass and grain yield were significantly affected by drought imposed at tillering and grain filling stages. The reduction in all these parameters have been reported previously by Aslam et al.^56^ and Muhammad et al.^57^ in quinoa and wheat under drought conditions, respectively. This reduction might be attributed to decreased plant metabolic functions, poor absorption and distribution of photosynthates under limited water supply^58^. The decreased NSPS, NGS, 1000 grain weight, biomass and yield under drought conditions was significantly improved by ZnO-NPs (150ppm), which might be due to the increased water and nutrient absorption, osmolytes accumulation and enhanced photosynthetic efficiency. This increase aligns with the findings of Dimkpa et al.^33^ and Abbas et al.^38^ in sorghum and wheat, respectively, which supports our results. However, Wang et al. reported a decrease in growth, gas exchange parameters, and biomass production in tomato plants at higher concentrations of ZnO-NPs (400 and 800 mg dm^− 3^). Furthermore, it has been reported that higher concentrations of ZnO-NPs can have toxic effects on crops leading to reduced growth, chlorophyll contents, stomatal conductance, and photosynthetic rate, ultimately reducing the biomass and yield^65,66^. The PCA biplot discovered several significant factors, including leaf chlorophyll contents, SC, Tr, and Pn as important descriptive variables enhancing the resilience of wheat to drought, aligning with the results of Raza et al.^58^ (Fig. 5).

## Conclusions

The current study revealed that varying dose levels of ZnO-NPs produced different effects on growth, yield, morpho-physiological traits, water relations, nutrient absorption, and grain quality parameters of wheat at critical growth stages (tillering and grain filling) under drought stress. Notably, the grain-filling stage was found as the most drought sensitive stage. Among the applied treatments, ZnO-NPs at 150 ppm showed the most promising effects in mitigating drought stress. The use of ZnO-NPs claimed an improvement in root shoot dimensions, plant dry biomass, grain yield, yield components, nutrient uptake, leaf relative water contents, gas exchange traits, chlorophyll concentration, grain zinc accumulation, and protein contents in drought subjected wheat at both tillering and grain filling stages. Since very little is known about the field-level application of ZnO-NPs, extensive field studies on various cereal crops under different drought exposures are required to validate their broader applicability.

## Data Availability

All data generated or analyzed during this study are included in this published article.
